# Relation of Macrophage Migration Inhibitory Factor to Pulmonary Hemodynamics and Vascular Structure and Carbamyl-Phosphate Synthetase I Genetic Variations in Pediatric Patients with Congenital Cardiac Shunts

**DOI:** 10.1155/2019/7305028

**Published:** 2019-02-06

**Authors:** Nair Y. Maeda, Vera D. Aiello, Paulo C. Santos, Ana M. Thomaz, Luiz J. Kajita, Sérgio P. Bydlowski, Antonio Augusto Lopes

**Affiliations:** ^1^Pró-Sangue Foundation, São Paulo 05403-000, Brazil; ^2^Heart Institute, University of São Paulo School of Medicine, São Paulo 05403-000, Brazil; ^3^EPM, Federal University of Sao Paulo (EPM-UNIFESP), São Paulo 04044-020, Brazil; ^4^LIM-31, University of São Paulo School of Medicine, São Paulo 05403-000, Brazil

## Abstract

Macrophage migration inhibitory factor (MIF) plays an important pathophysiological role in pulmonary hypertension (PHT). Previously, we demonstrated that serum MIF is increased in pediatric PHT associated with congenital heart disease (CHD). In the present study, we determined possible associations between MIF levels, hemodynamic and histological parameters, and mitochondrial carbamyl-phosphate synthetase I (CPSI) T1405N polymorphism in a similar population. The asparagine 1405 variant (related to A alleles in the C-to-A transversion) has been shown to be advantageous in pediatric PHT compared to the threonine 1405 variant (C alleles). Forty-one patients were enrolled (aged 2-36 months) and subsequently divided into 2 groups after diagnostic evaluation: the *high-pulmonary blood flow* (high PBF) group (pulmonary-to-systemic blood flow ratio 2.58 (2.21-3.01), geometric mean with 95% CI) and the *high-pulmonary vascular resistance* (high PVR) group (pulmonary vascular resistance 6.12 (4.78-7.89) Wood units × m^2^). Serum MIF was measured using a chemiluminescence assay. The CPSI polymorphism was analyzed by polymerase chain reaction followed by high-resolution melting analysis. Medial hypertrophy of pulmonary arteries was assessed by the histological examination of biopsy specimens. Serum MIF was elevated in patients compared to controls (*p* = 0.045), particularly in the high-PVR group (*n* = 16) (*p* = 0.022) and in subjects with the AC CPSI T1405N genotype (*n* = 16) compared to those with the CC genotype (*n* = 25) (*p* = 0.017). Patients with high-PVR/AC-genotype profile (*n* = 9) had the highest MIF levels (*p* = 0.030 compared with the high-PBF/CC-genotype subgroup, *n* = 18). In high-PVR/AC-genotype patients, the medial wall thickness of intra-acinar pulmonary arteries was directly related to MIF levels (*p* = 0.033). There were no patients with the relatively rare AA genotype in the study population. Thus, in the advantageous scenario of the asparagine 1405 variant (AC heterozygosity in this study), heightened pulmonary vascular resistance in CHD-PHT is associated with medial hypertrophy of pulmonary arteries where MIF chemokine very likely plays a biological role.

## 1. Introduction

Inflammation and immunity play a central role in the pathogenesis of pulmonary vascular disorders. A number of inflammatory mediators, such as cytokines, chemokines and their receptors, adhesion molecules and their ligands, components of the complement system, and proteases, were shown to play a pivotal role in the initiation and progression of pulmonary vascular abnormalities [1]. In pediatric pulmonary hypertension, mediators of inflammation were investigated in the human clinical setting and experimental models [2–4]. Studies have focused on specific etiologies of pulmonary arterial hypertension, as for the case of congenital heart disease [5, 6].

For chemokines, a role for the macrophage migration inhibitory factor (MIF) in clinical and experimental pulmonary vascular disease was demonstrated [1]. MIF is a noncanonical ligand of CXC chemokine receptors, leading to the activation of several signaling pathways that results in the inhibition of endothelial cell apoptosis and induction of smooth muscle cell proliferation. It is also associated with the exaggerated recruitment of peripheral blood mononuclear cells and proinflammatory endothelial cell behavior [1, 7–9]. In a recent study of ours involving pediatric patients with congenital cardiac communications, heightened serum MIF was observed in a specific subgroup of subjects with elevated pulmonary vascular resistance [10]. This finding suggests the possible involvement of this chemokine in the early development of pulmonary vasculopathy in congenital heart disease.

Another interesting observation in the pediatric population is the association of a functional polymorphism of the mitochondrial enzyme carbamyl-phosphate synthetase I (CPSI) with the development of pulmonary hypertension. A C-to-A nucleotide transversion in the exon 36 of the gene that encodes CPSI results in the substitution of threonine for asparagine at position 1405 (T1405N), an important cofactor binding site [11]. CPSI catalyzes the first step in the hepatic urea cycle in which arginine, the main substrate for nitric oxide production, is synthesized. Neonates with the AA T1405N genotype were shown to have higher circulating levels of arginine and nitric oxide metabolites [12]. In contrast to subjects with C alleles, neonates with at least one copy of the A allele (asparagine 1405 variant) were less likely to develop neonatal pulmonary hypertension [12]. Furthermore, the CPSI T1405N polymorphism was shown to be associated with postoperative pulmonary hypertension in children undergoing repair for congenital cardiac anomalies [13, 14].

Because of the functional role of the CPSI T1405N polymorphism in the pediatric population, particularly with regard to pulmonary circulation, we hypothesized that circulating levels of MIF may correlate with CPSI genotypes in young patients with congenital cardiac communications and altered pulmonary hemodynamics. Therefore, we examined possible correlations between serum MIF levels, patterns of clinical and hemodynamic presentation, and T1405N genotypes in these patients. A morphometric analysis of pulmonary vessels was performed to demonstrate the presence of arterial remodeling. We also examined the possible influence of Down syndrome.

## 2. Methods

### 2.1. Study Population

The study population consisted of pediatric patients (up to 3 years of age) who were admitted to the Heart Institute (InCor), University of São Paulo School of Medicine, Brazil, for repair of congenital cardiac communications. The presence of relatively simple cardiac anomalies with unrestrictive communication between the cardiac chambers and/or the great arteries (in the absence of pulmonary stenosis) was required for inclusion. Therefore, all patients had clinical features suggestive of elevated pulmonary arterial pressure. Patients with complex anomalies, including those with univentricular physiology, were not included. Neonates, patients under intensive care, and those with relevant comorbidities or extracardiac syndromes other than Down syndrome were not included. Having met the inclusion criteria, with an informed consent signed by family members, patients entered the study consecutively. After inclusion, patients were divided into 2 groups. Those with clinical features suggestive of pulmonary overcirculation (congestive heart failure with failure to thrive and an enlarged heart with pulmonary congestion) were considered for surgical treatment with no need for cardiac catheterization. Patients not presenting these features were assumed to have inappropriately elevated pulmonary vascular resistance and were assigned to cardiac catheterization to confirm this preliminary assumption. Mildly decreased peripheral oxygen saturation was observed in some of these patients, compatible with bidirectional shunting across the cardiac septal defects. Pediatric controls were recruited from the same geographic area and had the same distribution of ethnic backgrounds as the patients in the study. The control group contained subjects with Down syndrome as well, but with no signs of heart disease or pulmonary hypertension. They were screened at the Heart Institute. The study protocol was approved by the Institutional Scientific and Ethics Committee (CAPPesq no. 0502.11).

### 2.2. Echocardiography and Cardiac Catheterization

In addition to providing detailed anatomical data, transthoracic echocardiography was used for estimating the pulmonary-to-systemic blood flow ratio according to a previously reported methodology [15, 16]. The direction of flow across the cardiac defects and the size of the left cardiac chambers were also considered when assigning patients to one of the clinical groups. Left-to-right shunting (exclusive or predominant) in the presence of an enlarged left heart was considered as indicative of increased pulmonary blood flow. Bidirectional shunting with a relatively small-sized left heart was suggestive of heightened pulmonary vascular resistance. Cardiac catheterization was considered only for patients who were suspected to have higher levels of pulmonary vascular resistance based on clinical data and echocardiographic evaluation. Catheterization was performed under general anesthesia and mechanical ventilation. Pulmonary and systemic blood flow were determined using the Fick principle and used for the assessment of pulmonary and systemic vascular resistance [17]. The parameters were determined at baseline and during nitric oxide inhalation (40 ppm, 10 min).

### 2.3. Serum Levels of MIF

Peripheral venous blood was collected from patients and controls, and serum samples were obtained and stored at -80°C until use. After protein immobilization on nitrocellulose membranes, processing (immunoblotting) was performed as previously described [18], and MIF was detected using a human cytokine detection kit (R&D Systems, Minneapolis, MN, USA). Chemiluminescence was used for the semiquantitative assessment of MIF level in serum. The average signal of a pair of duplicate spots representing the MIF protein was normalized using internal controls, and the results are expressed as units of pixel intensity (upi).

### 2.4. CPSI T1405N Polymorphism

Genomic DNA from patients was extracted from peripheral blood using the salting-out procedure. Genotypes for the CPSI T1405N polymorphism were detected by polymerase chain reaction (PCR) followed by high-resolution melting (HRM) analysis with the Rotor Gene 6000® instrument (Qiagen, Courtaboeuf, France). The amplification of the fragments was performed using the following primers: sense 5′- AGCCACATCAGACTGGCTCA -3′ and antisense 5′- CTTCTTGAGACGGCCATGC -3′ (68 pairs base). A 35-cycle PCR was performed with an annealing temperature of 53.4°C. PCR was performed using a 10 *μ*L of reaction solution with the addition of fluorescent DNA-intercalating SYTO9® (1.5 *μ*M; Invitrogen, Carlsbad, CA, USA). In the HRM phase, the Rotor Gene 6000® measured the fluorescence for each 0.1°C temperature increase in the range of 68-80°C. The melting curve was generated by the decrease in fluorescence with the increase in temperature, and for the analysis, nucleotide changes result in different curve patterns.

### 2.5. Assessment of Medial Hypertrophy of Pulmonary Arteries

Lung biopsy specimens were collected in selected cases during cardiac surgery for the repair of heart lesions. Only patients with clinical features suggestive of inappropriately elevated pulmonary vascular resistance were considered for lung biopsy. The surgeon was asked to determine whether the procedure would be low risk on an individual basis. Specimens were collected with the airways distended, fixed in formalin, and subjected to histological processing. Four-micrometer-thick sections were obtained and subjected to hematoxylin-eosin stain and Miller's elastic stain. Arteries were landmarked by reference to the type of airway they accompanied, preacinar terminal bronchiolus, respiratory bronchiolus, and alveolar duct. The external diameter of an artery was measured between the external elastic laminae across the shorter axis of the vessel. Wall thickness was measured from the external to internal elastic lamina and computed as a percentage of the external diameter as follows:

(1) $$\begin{array}{c} \text{Percent wall thickness}=\frac{2\times \text{ wall thickness}}{\text{external diameter}}\times 100. \end{array}$$

In each patient, a mean value was calculated for each artery category, and the final result was obtained after a Z-score transformation. For this purpose, normal values for age were obtained from reference [19]. In average, 4 preacinar and 12 intra-acinar arteries were examined per patient.

### 2.6. Statistical Analysis

Results involving numeric variables are presented as geometric means with 95% confidence intervals (95% CI) or estimated marginal (adjusted) means with standard error (SE). For the categorical variables, data are presented as the number of patients and percentages or proportions. For most of the numeric variables corresponding to demographic and diagnostic findings, a normal distribution was not observed. Therefore, the differences between two groups were analyzed using the Mann-Whitney test. For categorical variables, the differences were analyzed using the chi-square family of tests. In all groups and subgroups, the MIF concentration distribution was sufficiently close to the normal distribution. Initially, the difference between patients and controls was tested using Student's *t*-test. However, MIF levels were influenced by age (linear regression analysis with calculation of Pearson's coefficient). For this reason, all subsequent analyses of MIF concentrations involving comparisons between groups were performed using the general linear model (analysis of covariance), including the age as a covariate, and the results are expressed as the adjusted means with SE. The analysis of covariance and regression analysis were used to test for possible associations between MIF levels and medial hypertrophy of pulmonary arteries and hemodynamic parameters. For all statistical procedures, 0.05 was adopted as the significance level. All tests were performed using the IBM SPSS statistical software (version 25, Armonk, NY, USA).

## 3. Results

Forty-one patients were enrolled, with ages ranging from 2 to 36 months. For the entire cohort, pulmonary-to-systemic blood flow ratio (echocardiography) was 2.29 (1.99-2.64) (geometric mean with 95% CI), and peripheral oxygen saturation was 94% (93%-96%). Twenty-seven patients (65.9%) had Down syndrome. Cardiac catheterization was considered necessary for the decision about treatments in 16 patients (high-pulmonary vascular resistance (PVR) group). The mean pulmonary arterial pressure was 54 (48-61) mmHg (normal levels, <25 mmHg), and the pulmonary vascular resistance was 6.12 (4.78-7.89) Wood units × m^2^ (normal levels, <3.00 U × m^2^). Patients for whom cardiac catheterization was considered unnecessary (high-pulmonary blood flow (PBF) group, *n* = 25) had a higher pulmonary-to-systemic blood flow ratio than did the high-PVR group (2.58 (2.21-3.01) and 1.88 (1.43-2.48), respectively, *p* = 0.024). Furthermore, they were younger (respective ages, 8.5 (6.5-11.1) mos. and 15.1 (10.8-21.2) mos., *p* = 0.009) and had higher peripheral oxygen saturation (96% (94%-97%) and 93% (91%-95%), respectively, *p* = 0.018). Thus, high-PBF and high-PVR groups were clearly distinct on the basis of clinical and hemodynamic parameters.

For the CPSI T1405N genotype distribution, only the genotypes CC and AC were present in the cohort (Table 1). The distribution of genotypes was skewed from the expected distribution within the general population (*p* = 0.010 compared with the Hardy-Weinberg equilibrium) [12]. However, it was not significantly different from the distributions reported in two studies involving children with postcardiac surgery pulmonary hypertension (*p* = 0.065 and *p* = 0.082 compared with the data reported in references [13] and [14], respectively).

Demographic, diagnostic, and hemodynamic variables in groups defined according to the CPSI T1405N genotypes present in the study population are depicted in Table 2. Lower levels of pulmonary arterial pressure and pulmonary vascular resistance were observed in patients with the AC genotype compared to those with the CC genotype. However, the differences must be interpreted with caution, as both variables are known to correlate directly with the patient's age. In the specific subgroup of patients subjected to cardiac catheterization (*n* = 16), subjects with the AC T1405N genotype were younger than those with the CC genotype (10.9 (7.1-16.6) and 21.1 (11.7-37.9) months of age, respectively, *p* = 0.051). No other differences were observed. In particular, there was no association of CPSI T1405N genotypes with the presence of Down syndrome.

**Figure 1:**
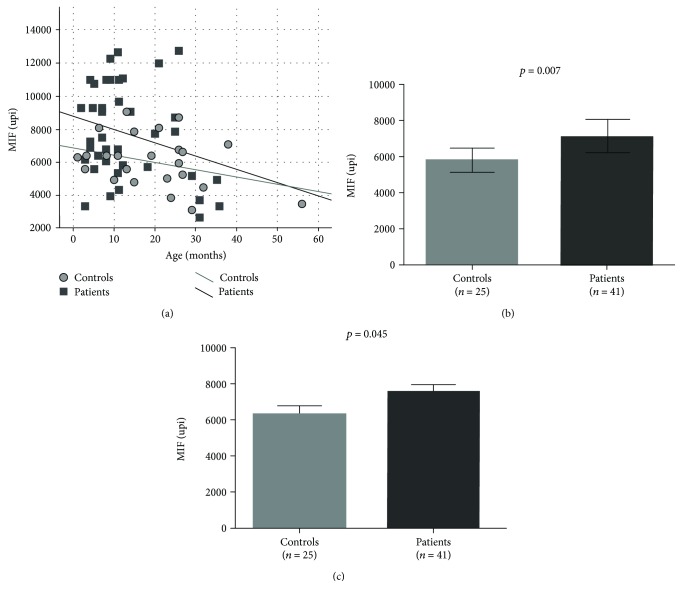
Serum MIF levels in patients compared to controls. (a) MIF levels tended to decrease with increasing age. Using analysis of covariance, *p* = 0.029 and *p* = 0.045, respectively, for the age effect and between-groups difference. For the whole study population, the equation was *y* = 8294.0 – 79.3^∗^x (*r* = −0.34), *p* = 0.005. On the bottom, MIF levels are shown as original values (b, unadjusted geometric means with 95% CI) and after adjustment for age (c, estimated marginal means with SE).

Serum MIF levels were influenced by the age in patients and pediatric controls (Figure 1). Levels were elevated in patients compared to controls even after adjustment for age. Figure 1 shows that the highest MIF levels were detected in young patients. In the figure, no controls are observed above the level of 10000 upi. MIF levels were not influenced by the presence or absence of Down syndrome (*p* = 0.180 for the entire study population, *n* = 66 and *p* = 0.581 for the patient population, *n* = 41).

**Figure 2:**
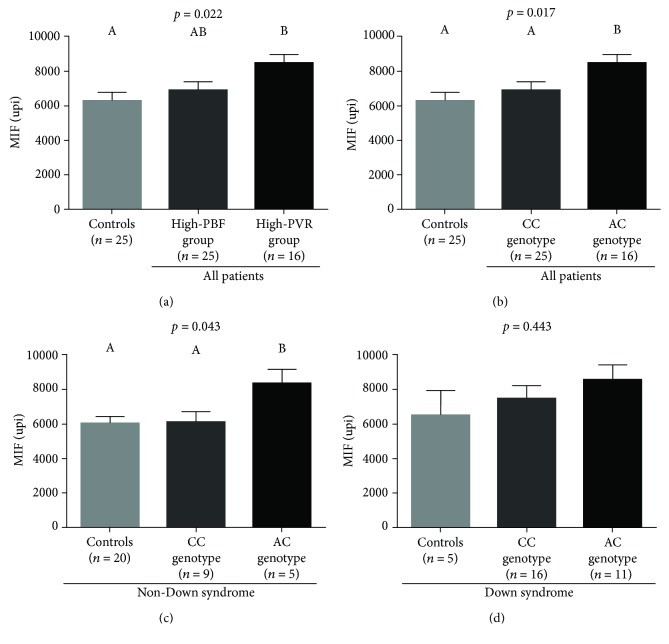
Serum MIF levels in patient groups according to the clinical/hemodynamic presentation (a) and carbamyl-phosphate synthetase I (CPSI) T1405N polymorphism (b, c, d). Despite a relatively small number of subjects for the subgroup analysis, specific data for the subgroups with (d) and without Down syndrome (c) are presented. In all graphs, the bars represent the estimated marginal means with SE after adjustment for age. Means not sharing the same letter were significantly different by *post hoc* multiple comparison tests. PBF and PVR, pulmonary blood flow and pulmonary vascular resistance, respectively. CC and AC, CPSI T1405N genotypes present in the study population.

**Figure 3:**
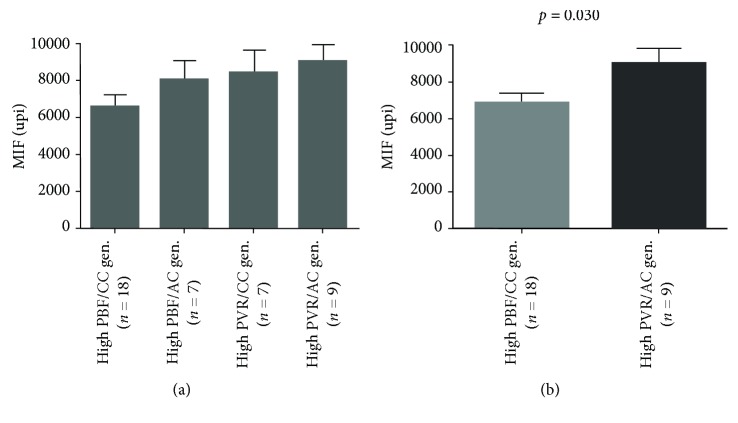
Serum MIF levels in patient subgroups according to the clinical/hemodynamic profile and CPSI T1405N genotypes analyzed in combination. All results are presented as estimated marginal means with SE after adjustment for age. PBF and PVR, pulmonary blood flow and pulmonary vascular resistance, respectively. CC and AC, CPSI T1405N genotypes present in the study population.

The analysis of serum MIF levels in patient groups according to the clinical and hemodynamic profiles and CPSI T1405N genotypes is shown in Figure 2. The highest levels were observed in the high-PVR group and in patients with the AC genotype compared to those with the CC genotype and controls. Figure 2 also shows that the pattern of MIF concentrations in controls and patient groups according to the CPSI genotypes did not seem to be influenced by the presence or absence of Down syndrome, acknowledging that there was a restricted power for subgroup analyses.  Figure 3 shows the levels of the MIF chemokine when the two factors (clinical/hemodynamic profile and CPSI T1405N genotypes) were analyzed in combination. Because of the small number of cases per subgroup, only one difference was tested. High-PVR/AC-genotype subjects had higher levels of MIF in the serum than those in the high-PBF/CC-genotype subgroup.

**Figure 4:**
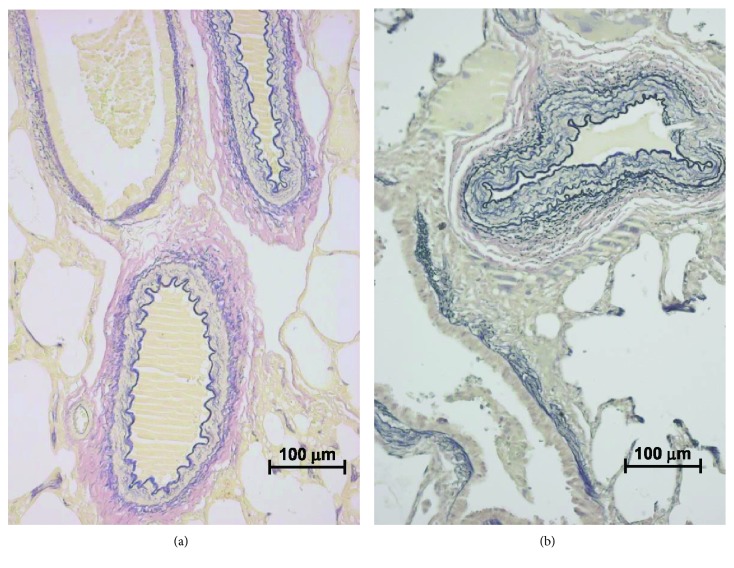
Photomicrographs of arteries accompanying terminal bronchioles showing severe hypertrophy of the medial layer and absence of intimal lesions. Miller's elastic stain, objective magnification 20x for both panels. (a) 9-month-old patient with Down syndrome, with atrioventricular septal defect. Peripheral oxygen saturation was 88%, and pulmonary vascular resistance was 5.3 Wood units × m^2^ decreasing to 1.8 units × m^2^ during nitric oxide inhalation. Z-score transformed pulmonary artery wall thickness was 8.1, and serum MIF level was 12305 upi by chemiluminescence. (b) 4-month-old patient, with ventricular septal defect. Peripheral oxygen saturation was 95%, and pulmonary vascular resistance was 4.0 Wood units × m^2^ decreasing to 1.3 units × m^2^ while on nitric oxide. Pulmonary artery wall thickness was 21.2 (Z-score), and serum MIF level corresponded to 11014 upi. Both patients had AC CPSI T1405N genotype.

**Figure 5:**
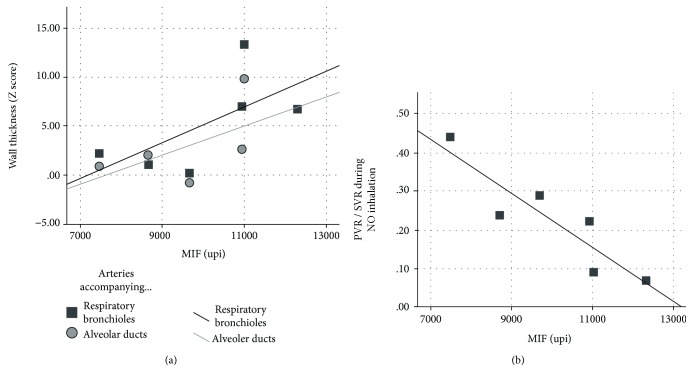
(a) Relation of medial hypertrophy of intra-acinar pulmonary arteries to the circulating (serum) levels of MIF chemokine. The data correspond to patients with high-PVR clinical profile and AC CPSI T1405N genotype for whom lung biopsy specimens could be collected intraoperatively (no significant association was seen in subjects with the CC genotype). These patients were characterized by high levels of serum MIF (i.e., above 7000 upi). The magnitude of wall thickness was similar between intra-acinar arteries (*p* = 0.470) but varied across MIF levels (*p* = 0.033). The regression equations for arteries accompanying the respiratory bronchioles and alveolar ducts were *y* = −12.99 + 1.81E-3^∗^x (*r* = 0.64), and *y* = −11.24 + 1.48E-3^∗^x (*r* = 0.66), respectively. (b) Pulmonary vascular response to inhaled nitric oxide (NO) in the same patients. The level of pulmonary vascular resistance at 10 minutes of NO inhalation is shown as pulmonary-to-systemic vascular resistance ratio (PVR/SVR). Subjects with higher levels of MIF (the ones with higher degree of arteriolar hypertrophy) had a more pronounced response (*y* = 0.92 – 6.98E-5^∗^x (*r* = −0.90), *p* = 0.014). No significant correlation was seen in patients with the CC genotype.

Lung biopsy material was available for the analysis of arterial wall thickness in 10 of 16 patients of the high-PVR group. Medial hypertrophy was present in arteries accompanying the terminal bronchioles, respiratory bronchioles, and alveolar ducts (Figure 4). The respective Z-scores were 7.28 (3.96-13.39), 3.53 (1.28-9.68), and 2.73 (0.91-8.19). A positive association was observed between serum MIF levels and the magnitude of medial hypertrophy of pulmonary arteries in 6 patients with the AC CPSI T1405N genotype (they were, in fact, high-PVR/AC-genotype subjects). However, the association was significant only for the arteries accompanying respiratory bronchioles and alveolar ducts (Figure 5). There was no similar trend in the high-PVR/CC-genotype patients (*n* = 4, data not shown). Pulmonary vascular occlusive lesions were not observed in any patients of the high-PVR/AC-genotype subgroup. Lesions were observed in 2 of 4 individuals in the high-PVR/CC genotype subgroup. Consistent with the association between MIF levels and pulmonary arteriolar hypertrophy was the observation that serum MIF correlated with pulmonary vascular response to inhaled nitric oxide. Again, this association was observed only in subjects with the AC genotype (Figure 5). Patients with high levels of MIF had a greater response, that is, low levels of PVR during nitric oxide inhalation.

## 4. Discussion

In this study, the elevated serum levels of MIF chemokine were observed in patients with congenital cardiac shunts and altered pulmonary hemodynamics compared to pediatric controls. The highest MIF levels were observed in younger patients, particularly those with a high-PVR presentation and were associated with the AC CPSI T1405N genotype. MIF was shown to stimulate pulmonary artery smooth muscle cell proliferation in hypoxic pulmonary hypertensive rats, an effect that involves ERK 1/2 and JNK phosphorylation [7]. In isolated pulmonary artery rings, MIF enhanced constriction in response to hypoxia and potentiated constrictions that were preevoked by agonists through the PKC, p38, and ERK 1/2 signaling pathways [20]. In another study, increased MIF in pulmonary arteries was associated with cyclin D1 upregulation via the ERK signaling pathway in pulmonary hypertensive broilers [21]. Considering that MIF is very likely involved in pulmonary vascular smooth muscle cell growth in human disease, the closeness between its levels and the CPSI T1405N polymorphism observed in this study suggests the existence of a phenotype in which some patients with heightened pulmonary vascular resistance (i.e., those with the asparagine 1405 variant) remain relatively protected from developing the most severe forms of the disease while sustaining a medial hypertrophy pattern of vascular remodeling. The association we observed between circulating MIF levels and pulmonary arterial wall thickness in high-PVR/AC-genotype patients is consistent with this hypothesis, recognizing the limitation of having a small number of lung biopsies for analysis in this study. Furthermore, heightened serum MIF correlated with the magnitude of pulmonary vasodilation in response to nitric oxide administration during cardiac catheterization.

We have only preliminary hypotheses about the possible relationships between MIF, the CPSI T1405N polymorphism, and pulmonary vasoreactivity in pediatric subjects. In the studies by Summar et al. [13] and Canter et al. [14], it was suggested that the postoperative elevation of pulmonary arterial pressure (i.e., patients with the CC CPSI genotype compared to those with the AA genotype) was due to increased pulmonary vascular tone. However, there were no specific data indicating an increased pulmonary vascular response to stimuli frequently present in the early postoperative period, such as changes in alveolar oxygen tension, pH, and pCO_2_. In the study by Pearson and coworkers involving neonates with pulmonary hypertension [12], vasoreactivity was not tested either. Obviously, patients in these studies had elevated pulmonary artery pressure, but it is not known whether they were vasoreactive. It is important to consider that pulmonary vasoreactivity and the pulmonary vascular response to vasodilators, for example, inhaled nitric oxide during preoperative cardiac catheterization in congenital heart disease, are not necessarily related phenomena. Furthermore, preoperative and postoperative contexts are considerably different and not comparable to the neonatal condition. The present study corresponds to preoperative observations. The data presented in Table 2 show that patients of both groups (CC and AC genotypes) had pulmonary vasodilation in response to inhaled nitric oxide. In particular, patients with the AC genotype had a mean value for pulmonary vascular resistance of <3.0 Wood units × m^2^ (considered the upper limit of normality) while on nitric oxide. These patients were also vasoreactive. This was suggested by a lower peripheral oxygen saturation at baseline than that in the subjects with the CC genotype (not a significant difference, *p* = 0.089), compatible with heightened pulmonary vascular resistance with some degree of right-to-left shunting across the cardiac septal defects. Furthermore, there was a higher proportion of patients with the high-PVR clinical profile in the AC-genotype group (*p* = 0.070). Vasoreactive patients may have some degree of pulmonary vasodilation at the beginning of cardiac catheterization, even before nitric oxide administration, as a result of anesthesia, mechanical ventilation, and muscle relaxation. This may partly explain the differences between groups with regard to pulmonary arterial pressure and pulmonary vascular resistance. Interestingly, patients with the AC genotype and a high-PVR clinical profile had the highest circulating levels of MIF, suggesting a role for this chemokine in pulmonary vasoreactivity.

In vascular and inflammatory processes, the relation of cytokine expression to arginine availability and nitric oxide production cannot be easily anticipated. As a rule of thumb, L-arginine and nitric oxide have important anti-inflammatory, antithrombotic, and antiproliferative properties [22]. However, in addition to being a substrate for nitric oxide production by nitric oxide synthetases, L-arginine can be metabolized to urea and L-ornithine by arginases. In fact, arginases and nitric oxide synthetases effectively compete for L-arginine. The product of arginase activity, L-ornithine, is a precursor for the production of polyamines and proline, which controls cell proliferation and collagen synthesis, respectively [23]. In experimental models, increased arginase I expression was shown to be associated with increased aortic smooth muscle cell and endothelial cell proliferation [24, 25]. In pediatric pulmonary hypertension, the CPSI T1405N polymorphism was studied with a focus on arginine and nitric oxide metabolite production [12–14], and not on the activation of arginase pathway and subsequent events. For nitric oxide itself, patients with more effective production (i.e., those with the asparagine 1405 CPSI variant) would be expected to be relatively protected from inflammatory insults. However, there have been conflicting data about the role of nitric oxide in the regulation of cytokine expression. Some studies indicate a proinflammatory role of nitric oxide [26, 27]. The immunoregulatory effects of nitric oxide seem to be determined by its levels, although the type of responder cell, the cytokine, and the stimulant also play a role in different experiments [28]. Nitric oxide was shown to induce MIF mRNA and protein release in human fetal membranes [29]. Additional observations may have implications for the results of the present study. For example, MIF can elicit the Th2-type immune response [30], shown to be involved in experimental pulmonary vascular remodeling [31], and arginase I is strongly induced by Th2 cytokines [32, 33].

In conclusion, data from the present study indicate the existence of a subset of young patients with congenital cardiac shunts in whom heightened circulating levels of MIF chemokine are associated with medial hypertrophy of small pulmonary arteries and elevated pulmonary vascular resistance. Some of our findings suggest that pulmonary vascular resistance is dynamic rather than fixed in these patients. In our study population, this phenotype was associated with AC heterozygosity for the CPSI T1405N polymorphism, consistent with previous observations that the presence of at least on copy of the A allele is protective against aggressive forms of pulmonary vascular disease. Our data do not provide evidence for any causal relationship between CPSI T1405N polymorphism and cytokine (MIF) expression. However, in theory, the existence of interrelated biological pathways is possible. Further studies are warranted for a better understanding of these interrelationships in the pediatric population.

## Figures and Tables

**Table 1:** CPSI T1405N genotype distribution in the study population.

	CC	AC	AA
General population^∗^, % (*n* = 460)	42.0	46.0	12.0
Study population, *n* (%) (*n* = 41)	25 (61.0)	16 (39.0)	0 (0.0)

^∗^According to reference [12]. CC denotes homozygosity for the C-encoded Thr 1405 variant; AA, homozygosity for the A-encoded Asn 1405 variant; and AC, heterozygosity for this polymorphism at position 1405.

**Table 2:** Demographic, diagnostic, and hemodynamic variables in groups defined according to the CPSI T1405N polymorphism.

	CPSI genotypes in the study		*p*
	CC (*n* = 25)	AC (*n* = 16)	
Age, months	9.9 (7.2-13.6)	10.4 (7.2-15.0)	0.726†
Gender, M : F	6 : 19	7 : 9	0.326‡
Down syndrome, *n* (%)	16 (64.0)	11 (68.8)	0.754‡
Peripheral oxygen saturation (%)	95 (94-97)	93 (91-95)	0.089†
Pulmonary-to-systemic blood flow ratio§	2.43 (2.01-2.94)	2.10 (1.65-2.66)	0.341†
High-PBF patients : high-PVR patients	18 : 7	7 : 9	0.070‡
Diagnosis^∗^			0.110ǁ
A, *n* (%)	1 (4,0)	0 (0.0)	
B, *n* (%)	16 (64.0)	6 (37.5)	
C, *n* (%)	7 (28.0)	10 (62.5)	
D, *n* (%)	1 (4.0)	0 (0.0)	
Cardiac catheterization	CC (*n* = 7)	AC (*n* = 9)	
Mean pulmonary arterial pressure (mmHg)	61 (54-70)	49 (41-58)	0.031†
Mean systemic arterial pressure (mmHg)	68 (61-77)	59 (51-68)	0.107†
Pulmonary vascular resistance (U × m^2^)	7.98 (4.88-13.07)	4.98 (3.98-6.24)	0.039†
Systemic vascular resistance (U × m^2^)	14.94 (11.83-18.85)	13.56 (11.00-16.71)	0.536†
Pulmonary vascular resistance while on inhaled nitric oxide (U × m^2^)	5.35 (2.13-13.41)	2.98 (2.03-4.39)	0.114†

Numeric variables are expressed as geometric mean (95% CI). ^†^Mann-Whitney test; ^‡^Chi-square test; ^ǁ^likelihood ratio; ^§^estimated by echocardiography. ^∗^A: pretricuspid defects, B: posttricuspid defects except for atrioventricular septal defects, C: atrioventricular septal defects, D: conotruncal defects. CPSI: carbamyl-phosphate synthetase I.

## Data Availability

The data used to support the conclusions of the present study correspond to the projects FAPESP #2011/09341-0 and 2015/21587-5 and are available from the corresponding author upon request.
